# Enzyme promiscuity powers plant chemical diversity: A case of prenyltransferases in biosynthesis of quinone derivatives

**DOI:** 10.1093/plphys/kiae227

**Published:** 2024-04-23

**Authors:** Ryo Yokoyama

**Affiliations:** Assistant Features Editor, Plant Physiology, American Society of Plant Biologists; Max-Planck-Institute of Molecular Plant Physiology, Am Mühlenberg 1, Potsdam-Golm 14476, Germany

Plants are capable of producing between 200,000 and 1 million natural products (specialized metabolites). Such chemodiversity has emerged, occasionally in a lineage-specific manner, as plants adapted to challenging environmental niches on land (Wang et al. 2019; Maeda and Fernie 2021). How has phytochemical diversity evolved? How do plants develop a new biosynthetic pathway for specialized metabolites? These fundamental questions have been studied using genomics, biochemistry, and metabolomics, with the potential for metabolic engineering in synthetic biology platforms (Maeda 2019).

Enzymes catalyze chemical reactions that convert specific substrates into products. While enzyme-substrate matching is generally assumed to be highly specific, some enzymes can react with multiple substrates. This broad substrate specificity is often referred to as “enzyme promiscuity” and is considered to be one of the major driving forces behind the generation of new enzyme functions (neofunctionalization) (Khersonsky and Tawfik 2010). A growing body of evidence suggests that substrate promiscuity allowed ancestral enzymes to have the capacity for neofunctionalization with fewer genetic changes, leading to the emergence of new metabolic pathways. In other words, there may be evolutionary pressure to maintain enzyme promiscuity to be prepared for future metabolic expansion in plant specialized metabolism (Pichersky and Lewinsohn 2011; Weng et al. 2012).

In this issue of *Plant Physiology*, Liu et al. identified a novel enzyme catalyzing the committed step in the biosynthesis of quinone-derived specialized metabolites, providing a clear example of enzyme evolution that contributes to plant chemodiversity (Liu et al. 2024). Quinone is one of the aromatic compounds with various biological functions, such as ubiquinone for electron carriers in mitochondria and phylloquinone (vitamin K) for cofactors (Symons 2021). In addition, some plant species produce and accumulate pharmaceutical quinone derivatives in a lineage-specific manner. For example, anthraquinones form the largest group of quinone derivatives produced in Rubiaceae and are widely used as natural dyes and pharmaceuticals (Wang et al. 2023). Shikonin is a naphthoquinone abundant in traditional medicinal plant species belonging to the Boraginaceae family (Yadav et al. 2022).

In plants, the main anthraquinone and naphthoquinone biosynthetic routes are commonly initiated with the conversation of shikimate, a key intermediate in aromatic amino acid biosynthesis, into 1,4-dihydroxy-2-naphthoic acid (DHNA) and 4-hydroxybenzoic acid (PHBA), followed by the addition of a prenyl group to DHNA and PHBA, respectively. 3,3-dimethylallyl pyrophosphate, derived from the methylerythritol phosphate (MEP) pathway, acts as a prenyl donor in the DHNA prenylation of anthraquinone biosynthesis (Han et al. 2002). DHNA prenylation is important for anthraquinone biosynthesis, as it is located at a metabolic confluence point between aromatics production and the MEP pathway. However, a prenyltransferase enzyme responsible for DHNA prenylation in the Rubiaceae-specific anthraquinone biosynthesis has yet to be elucidated.

**Figure 1.**
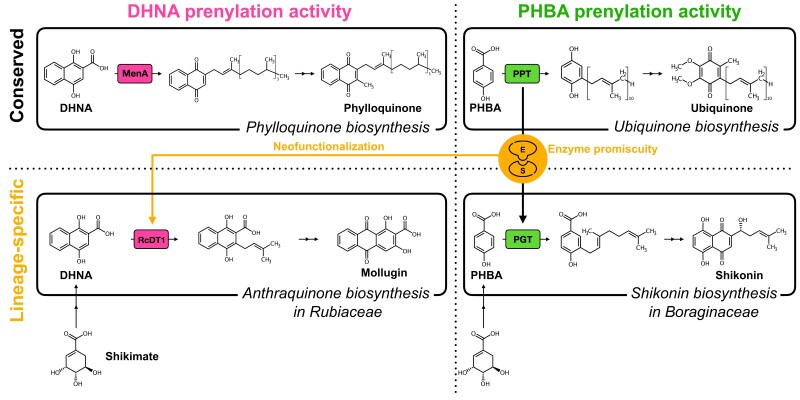
The enzyme promiscuity-driven evolutionary trajectory of DHNA and PHBA prenylation activities in the UbiA protein family. Enzyme promiscuity of the UbiA protein family was likely a driving force for the convergent evolution of DHNA prenylation activity of RcDT1 from the ubiquinone biosynthetic enzyme. This neofunctionalization of new prenylation activity contributed to lineage-specific chemical diversification of quinone derivatives. Mollugin was chosen as a representative anthraquinone in this figure.

Liu et al. conducted an activity-guided homology search using an RNA-seq library of *Rubia cordifolia* anthraquinone-rich cell suspension culture. Among 6 candidate genes, the authors identified a gene, *Rubia cordifolia dimethylallyltransferase 1* (*RcDT1*), encoding a prenyltransferase that belonged to the UbiA enzyme superfamily. In vitro enzymatic characterization of recombinant RcDT1 microsomes and in vivo RNA interference of the *RcDT1* gene in *R*. *cordifolia* callus cells validated that RcDT1 was a prenyltransferase mediating the DHNA prenylation. Strikingly, this study not only reported the identification of an enzyme involved in a plant specialized metabolic pathway but also highlighted the unique evolutionary trajectory of the UbiA enzyme family that contributed to lineage-specific chemodiversiy. RcDT1 and its homologs in other Rubiaceous plants reacted not only with DHNA but also with PHBA, an intermediate in ubiquinone and shikonin (naphthoquinone) biosynthesis. The promiscuous recognition of DHNA and PHBA indicates that DHNA and PHBA prenyltransferases share a common evolutionary origin. A phylogenetic tree of plant aromatic prenyltransferases in the UbiA family demonstrated that RcDT1 and its homologs were evolutionarily closer to *p*-hydroxybenzoate geranyltransferase (PGT), a key PHBA-prenylation enzyme in naphthoquinone biosynthesis, and PHBA polyprenyltransferase (PPT), a highly conserved enzyme in ubiquinone biosynthesis. On the other hand, RcDT1 and its homologs diverged from MenA enzymes, which prenylate DHNA for phylloquinone biosynthesis. With this clear evolutionary separation of 2 enzymes reacting with DHNA, the findings by Liu et al. suggest that RcDT1 convergently evolved via recruitment from the ubiquinone biosynthetic pathway but independently from prenyltransferases in phylloquinone biosynthesis (Fig. 1).

The promiscuous substrate recognition in the UbiA enzyme family is likely one of the critical factors for the convergent evolution of DHNA-prenylation activities for anthraquinone and phylloquinone biosynthesis. Similar convergently evolved neofunctionalization events have been reported in other UbiA family proteins, including coumarin *C*-prenyltransferases in Apiaceae and Moraceae (Munakata et al. 2020), coumarin *O*-prenyltransferases in Apiaceae and Rutacea (Munakata et al. 2021), and flavonoid and stilbene prenyltransferases in Moraceae and Fabaceae (Zhong et al. 2018). At the polypeptide sequence level, however, it remains to be seen how these prenyltransferases acquire new enzymatic activities. Phylogeny-guided structure-function analysis of multiple target enzymes across lineages will assist in the identification of amino acid residue(s) that are responsible for DHNA activities convergently emerging in anthraquinone and phylloquinone biosynthesis. These natural genetic innovators that change enzymatic activities and redirect metabolic pathways will be game-changers in plant synthetic biology to synthesize high-value chemicals at a large scale (Maeda 2019; Schenck and Busta 2022).
